# Maternal Diet Modulates Placenta Growth and Gene Expression in a Mouse Model of Diabetic Pregnancy

**DOI:** 10.1371/journal.pone.0038445

**Published:** 2012-06-11

**Authors:** Claudia Kappen, Claudia Kruger, Jacalyn MacGowan, J. Michael Salbaum

**Affiliations:** 1 Department of Developmental Biology, Pennington Biomedical Research Center, Louisiana State University System, Baton Rouge, Louisiana, United States of America; 2 Laboratory of Regulation of Gene Expression, Pennington Biomedical Research Center, Louisiana State University System, Baton Rouge, Louisiana, United States of America; Instituto Gulbenkian de Ciência, Portugal

## Abstract

Unfavorable maternal diet during pregnancy can predispose the offspring to diseases later in life, such as hypertension, metabolic syndrome, and obesity. However, the molecular basis for this phenomenon of “developmental programming” is poorly understood. We have recently shown that a diet nutritionally optimized for pregnancy can nevertheless be harmful in the context of diabetic pregnancy in the mouse, associated with a high incidence of neural tube defects and intrauterine growth restriction. We hypothesized that placental abnormalities may contribute to impaired fetal growth in these pregnancies, and therefore investigated the role of maternal diet in the placenta. LabDiet 5015 diet was associated with reduced placental growth, commencing at midgestation, when compared to pregnancies in which the diabetic dam was fed LabDiet 5001 maintenance chow. Furthermore, by quantitative RT-PCR we identify 34 genes whose expression in placenta at midgestation is modulated by diet, diabetes, or both, establishing biomarkers for gene-environment interactions in the placenta. These results implicate maternal diet as an important factor in pregnancy complications and suggest that the early phases of placenta development could be a critical time window for developmental origins of adult disease.

## Introduction

Maternal diet has long been known to be a key determinant for pregnancy success. Both undernutrition and malnutrition are harmful to development of the conceptus, increasing risk for spontaneous abortions, congenital malformations, and intrauterine growth restriction [1], [2]. However, it is now becoming clear that overnutrition and excess of particular nutrients, such as with maternal obesity or diabetes, are also detrimental [3], [4], [5], [6].

Unfavorable maternal diet, as reflected in abnormal birth weight, is believed to predispose the offspring to diseases later in life, such as hypertension, metabolic syndrome, and obesity [7], [8]. However, it is currently unclear which tissue systems are involved in this phenomenon of “developmental programming”. Using a mouse model of diabetic pregnancy, we have recently shown that a diet nutritionally optimized for pregnancy can nevertheless be harmful [9]. In the context of maternal hyperglycemia, this diet interacts with maternal metabolic conditions, leading to a more than three-fold increased rate of neural tube defects compared to occurrence of these defects when the pregnant diabetic dam is fed a maintenance chow [9]. Under these adverse conditions even embryos that were not obviously malformed were nonetheless negatively affected, as demonstrated by reduced fetal growth. In particular, while the two diets had similar carbohydrate content, higher fat content at the expense of protein content (while not protein-deficient) reduced fetal growth by 18%, and reduced size was already evident as early as gestational day E9.5, whereas higher protein and lower fat content reduced fetal growth by 9.3%, and only in late gestation [9]. Although the diets also differed in some other micronutrient components, these results are consistent with the notion that macronutrient composition of the maternal diet modulates the extent of intrauterine growth restriction in diabetic pregnancies. The impaired fetal growth clearly suggested that nutrient supply to the fetus was impaired, and that the placenta, the major conduit of nutrients to the fetus, might also be compromised.

Indeed, as we reported previously, placental growth was also reduced in pregnancies affected by maternal hyperglycemia [10]. In addition, we found abnormal cell differentiation and altered gene expression in the diabetic placenta as early as midgestation stages in the mouse. This suggested that, similar to adverse effects on the embryo (malformation) and fetal growth, the unfavorable maternal diet might also have detrimental effects on placental development. The present study tested this hypothesis using two diets, Purina 5001 (LabDiet 5001), a commercial rodent maintenance chow, and Purina 5015 (LabDiet 5015), a commercial diet specifically recommended for pregnancy and lactation; for ease of reading, we will refer to 5001 as “chow”, and 5015 as “breeder diet”. Our results identified diet-responsive genes in the murine placenta and their interaction with maternal diabetes. These genes may play a role not only for diet-induced placental impairment but also in developmental programming.

## Results

**Figure 1:**
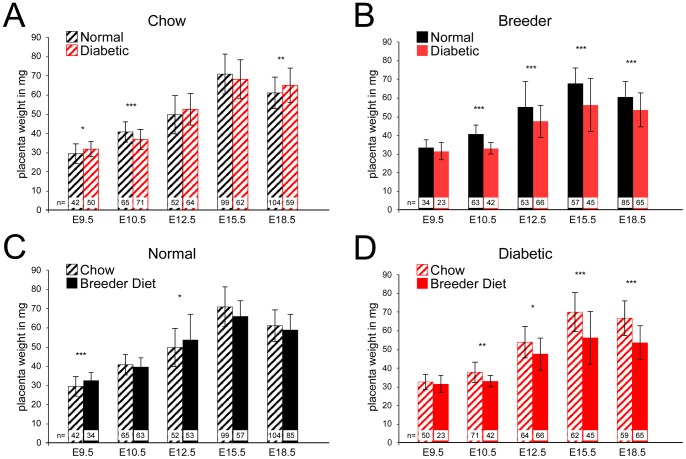
Interaction of maternal diet and maternal diabetes in placenta growth. Placentas were isolated at various time points; the dissected material consisted of both embryo-derived and maternal portions. Only placentas were used that were associated with morphologically normal embryos. Wet weight was determined immediately after dissection. n = number of placenta samples. Error bars show standard deviations from the means. A: Comparison of placenta weights between control and diabetic pregnancies where all dams were fed chow diet. B: Comparison of placenta weights between control and diabetic pregnancies where all dams were fed breeder diet. C, D: Replotted data from A and B to facilitate comparison by diet. *: p<0.05; **: p<0.005; ***: p<0.0005. In diabetic dams fed chow diet, placenta weights were indistinguishable from controls by the end of pregnancy, while in diabetic dams fed breeder diet, placentas were consistently smaller than controls after midgestation.

In order to investigate the effects of maternal diet on the placenta in diabetic pregnancies, we used the well-established STZ-induced diabetes FVB mouse model [9], [10], [11]. Females were fed either chow or breeder diet for at least 4 weeks before the first STZ treatment, and were considered diabetic when their blood glucose levels exceeded 250 mg/dL. They were then mated to non-diabetic FVB males, and placentas were isolated at various stages of the pregnancy and wet weight was determined (Figure 1). Only placentas associated with morphologically normal embryos were used for this study.

### Effect of maternal diet on placenta growth in diabetic pregnancies

When the dams were fed chow diet, differences between normal and diabetic placentas were observed at midgestation (E9.5, E10.5), and at the end of pregnancy (E18.5). Although statistically significant, the magnitude of weight differences was small: under 10% (recalculated to the normal weight average), and directions of change -i.e. increase/decrease- were not consistent between time points (Figure 1, Panel A). In contrast, when dams were fed breeder diet, the placentas in diabetic dams were consistently smaller than in normal pregnancies (Figure 1, Panel B). Also, the differences were generally greater than 10%, with up to 18% at E18.5. Thus, maternal diabetes during pregnancy is associated with reduced placenta growth when the breeder diet is consumed.

The breeder diet has little effect on placenta growth in normal pregnancies, although, compared to chow, significantly higher placental weights were observed at E9.5 (14.8% increased weight) and on E12.5 (13% increased weight) (Figure 1, Panel C). Thus, the breeder diet, which contains more fat, may promote placenta growth some stages. In contrast, in the context of a diabetic pregnancy (Figure 1, Panel D), the breeder diet is consistently associated with significantly lower placenta weight from E10.5 on, through late stages of pregnancy. In these diabetic pregancies, placenta weight was reduced by ∼8% at E10.5 and E12.5, and up to ∼15% at E15.5 and E18.5 (calculated relative to the chow diet). Thus, reduced placenta growth with breeder diet is due to a diet x diabetes interaction.

**Figure 2:**
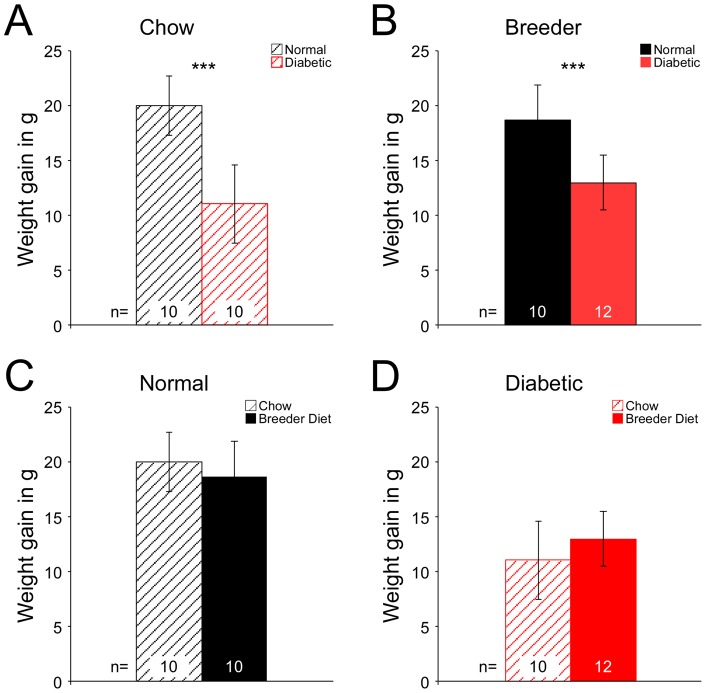
Effects of diet and diabetes on maternal weight gain. Pregnant females were weighed on the day the copulation plug was detected (E0.5), and before sacrifice. Weight gain was calculated as the difference between weight on E18.5 and E0.5. Bar diagrams depict means and standard deviations. A: On chow diet, diabetic dams gain less weight (p = 5.69x10^−6^ versus non-diabetic dams). B: On breeder diet, diabetic dams also gain less weight (p = 1.5x10^−4^ versus non-diabetic dams on breeder diet). C, D: Replotted data from A and B to facilitate comparison by diet. Differences between group means in C and D, respectively, are not statistically significant.

This conclusion is further supported when we consider placenta weights relative to maternal weight during pregnancy (Figure 2). Diabetic dams had reduced weight gain compared to controls on both diets (Panels A and B). However, there was no significant influence of diet on weight gain when either control (C) or diabetic dams (D) on the different diets were compared. Thus, the reduction in maternal weight gain is entirely due to the effects of maternal diabetes, diet had no significant effect. Therefore, maternal weight alone cannot explain the reduced placenta growth in diabetic dams that are fed breeder diet.

**Figure 3:**
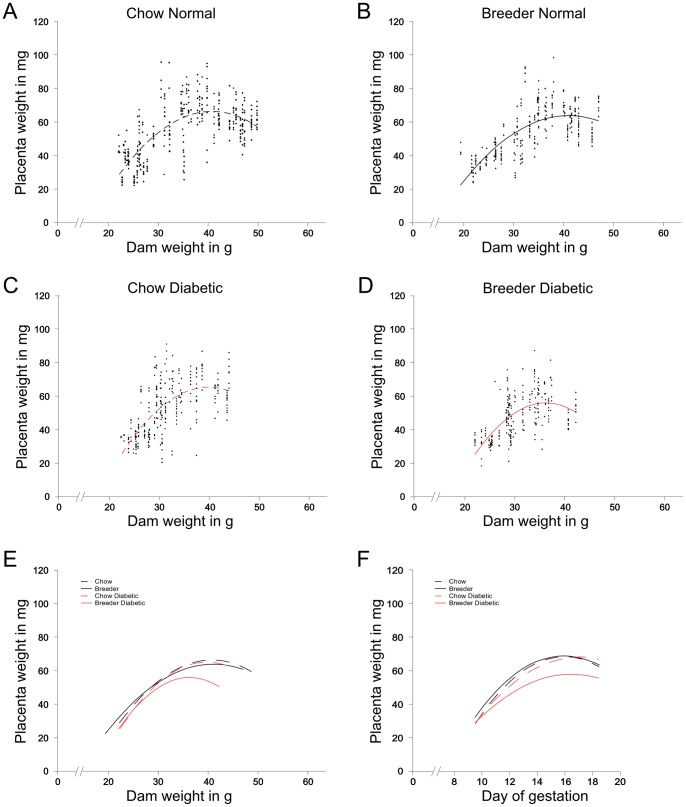
Relationship of placenta size to maternal weight – interaction of diet and diabetes. For each pregnant dam, her weight at sacrifice (timepoints from E9.5 to E18.5) was plotted along the X-axis, and the weights of placentas associated with morphologically normal embryos from her pregnancy were plotted along the Y-axis. Polynomial distributions were fitted to the data for A: Control dams fed chow diet; B: Control dams fed breeder diet; C: Diabetic dams fed chow diet: and D: Diabetic dams fed breeder diet. E: Comparison of the polynomial curves shows that the distribution of placenta weights during the pregnancy is very similar for all groups, except for the group of diabetic dams that were fed breeder diet. F: Comparison of polynomial distributions when the data are plotted by day of placenta isolation. Again, the results for diabetic dams on breeder diet reveal a specific interaction between diet and diabetes on placenta growth.

The relationship of placenta size to maternal weight is depicted in Figure 3: maternal weight at time of sacrifice is plotted along the x-axis, with all placenta weights for that individual's pregnancy plotted vertically along the y-axis. The distributions for normal pregnancies are very similar between chow-fed and breeder-diet-fed dams in both dimensions; accordingly, the shapes of the polynomial curves that describe the data are very similar as well. In contrast, peak maternal weights in diabetic pregnancies remain lower (compare the extension of data points along the x-axis between panels A-C and B-D, respectively). While the shape of the polynomial curve for placentas from diabetic chow-fed dams resembles that of normal pregnancies, the curve for diabetic pregnancies under conditions of breeder diet is clearly distinct (Figure 3E); with a reduction in height relative to the y-axis. This is also the case when placenta weights are plotted relative to day of dissection (Figure 3F). The placentas from diabetic dams fed the breeder diet substantially fail to expand during the second half of the pregnancy. This indicates that, although the effects of the diet x diabetes interaction on placenta growth are most pronounced in late pregnancy, underlying mechanisms controlling growth must already have been affected at midgestation. This proposition provided the rationale to investigate the effects of maternal diet on gene expression in the diabetic placenta at the midgestational E10.5 time point.

### Effect of maternal diet on placental gene expression in diabetic pregnancies

**Figure 4:**
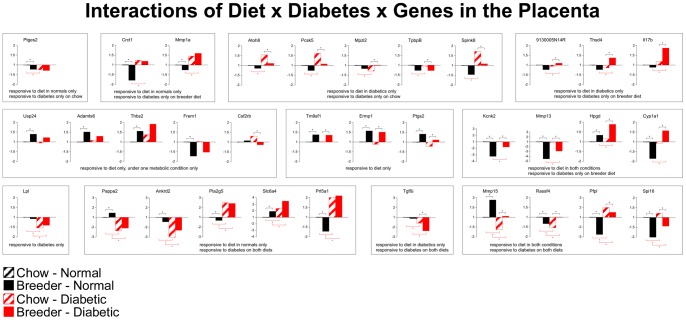
Interaction of maternal diet and diabetes on gene expression in the placenta. For each experimental group, gene expression levels were determined by quantitative real-time PCR (see Table 3). From the ΔC_t_-values, “fold-change“ of expression levels was determined relative to the expression level in placentas from normal dams on chow diet. * indicates that the difference between groups is statistically significant. Note that the Y-axis dimensions are different for some panels.

We previously published that, under conditions of maternal diabetes, placental gene expression is dysregulated by E10.5, labyrinth and junctional layer are reduced, and spongiotrophoblast migration is aberrant [10]. From this evidence and the literature, we selected 34 genes according to the following criteria: detectable expression in both control and diabetic samples, significant fold-change between these conditions, novelty of detection of a given gene in placenta, annotation for roles in metabolism, cell migration and proliferation, or on the basis of known cell specificity. For some of these genes, in fact, our results are the first to demonstrate expression in the placenta. Figure 4 displays the expression levels of each gene in control (normal pregnancies) and diabetic placentas for pregnancies where the dams were fed either chow or breeder diet, respectively (each group consisted of 6 samples from independent pregnancies; n = 6). For ease of visualization, “fold-change” of expression differences from the level in the normal/chow condition (set to 1) was plotted.

The results revealed multiple modes in which diet and diabetes affected gene expression in the placenta at E10.5: *Ptges2* (encoding prostaglandin E synthase 2) was responsive to diet only in normal dams, such that breeder diet was associated with lower expression. When chow diet was consumed, expression of this gene was responsive to diabetes, resulting in lower expression; breeder diet did not further reduce its level in diabetic dams. While significant in two comparisons, the magnitude of changes for this gene was rather moderate. *Crct1* (cysteine-rich C-terminal protein 1) and *Mmp1a* (matrix metallopeptidase 1a, interstitial collagenase) expression levels were also modulated by diet only in normal pregnancies, but diabetes had an effect in dams fed the breeder diet. *Atoh8* (atonal homolog 8), *Pcsk5* (proprotein convertase subtilisin/kexin type 5), *Mpzl2* (myelin protein zero-like 2), *TpbpB* (trophoblast specific protein beta), and *Spink8* (serine peptidsase inhibitor, Kazal type 8) were responsive to diet only under conditions of maternal hyperglycemia, and responsive to diabetes only under chow feeding conditions. Conversely, *9130005N14R*, *Thsd4* (thrombospondin type 1 domain containing 4), and *Il17b* (interleukin 17B) were also responsive to diet only under conditions of hyperglycemia, but then responded to diabetes when breeder diet was consumed. In each of these subsets, response to diet or diabetes was restricted to one modality only. This was also found for one group of genes that were not responsive to diabetes at all but solely to diet. *Usp24* (ubiquitin specific peptidase 24), *Adamts6* (ADAM metallopeptidase with thrombospondin type 1 motif 6), *Thbs2* (thrombospondin 2), and *Frem1* (Fras1 related extracellular matrix protein 1) exhibited diet response only in normal dams, and *Csf2rb* (colony stimulating factor 2 receptor beta) only in diabetic dams. It is interesting to note that the directions of changes in expression levels were diverse for different genes within each subset. However, for the three genes that respond only to diet regardless of metabolic condition, i.e. *Tm9sf1* (transmembrane 9 superfamily member 1), *Ermp1* (endoplasmic reticulum metallopeptidase 1), and *Ptgs2* (prostaglandin-endoperoxide synthase 2), breeder diet was always associated with increased expression levels. Elevated and reduced expression levels were found for the group of genes that respond to diet under any condition but to diabetes only with breeder diet, constituted by the *Kcnk2* (potassium channel subfamily K member 2), *Mmp13* (matrix metallopeptidase 13), *Hpgd* (hydroxyprostaglandin dehydrogenase 15), and *Cyp1a1* (cytochromome P450 family 1 subfamily a polypeptide 1) genes. *Lpl* (lipoprotein lipase) was not the only gene that responded to diabetes only regardless of diet; a larger study of diabetes-regulated genes will be published elsewhere [Kruger et al., unpublished results]. *Pappa2* (pappalysin 2), *Ankrd2* (ankyrin repeat domain 2), *Pla2g5* (phospholipase A2 group V), *Slc6a4* (solute carrier family 6 member 4, serotonin transporter), and *Prl5a1* (prolactin family 5 submaily a member 1) levels were also altered by maternal hyperglycemia, but diet had an effect only in normal pregnancies. In contrast, *Tgfßi* (transforming growth factor beta induced), while responsive to hyperglycemia, responded to diet only in the diabetic condition.

Finally, the group comprised of *Mmp15* (matrix metallopeptidase 15), *Rassf4* (ras association domain family member 4), *Pfpl* (pore forming protein-like), and *Spi16* (serine protease inhibitor 16) responded to both diabetes and diet, in all combinations. Notably, while the pattern of response may be similar for genes within one subset, the direction of change by diet, or by diabetes, can be different: for example, among the genes responsive to diet in both metabolic states but to diabetes only with breeder diet, *Kcnk2* and *Mmp13* expression levels are reduced by breeder diet, and increased by diabetes with the breeder diet. In contrast, *Hpgd* expression is increased by breeder diet, and also increased by diabetes. An even more complex response is exhibited by *Cyp1a1*, which displays antipodal responses in normal compared to diabetic conditions: the breeder diet reduces expression in placentas from normal dams, but increases expression in placentas from diabetic dams. These complex patterns of changes in gene expression levels suggest that within a given subset of genes that have in common responsiveness to diet, or diabetes, or both, different mechanisms exist for their regulation by the metabolic and diet conditions.

It is noteworthy that, even though the selection of genes investigated here was not random, almost all possible responses, based upon statistical significance of changes, were reflected in our data: with comparisons between 4 conditions, 16 response patterns are theoretically possible (see Table 1). We are not considering patterns 1 (no response at all), 4 and 5 (response to diabetes in only one or the other diet condition) here, since data for these have been published previously [10]. Intriguingly, of the remaining 13 possibilities, 12 patterns were represented in our results, 8 by more than one gene. We currently do not know whether the one undetected possibility (pattern 12) could be represented in a larger gene set, or whether there are biological reasons for the absence. Nonetheless, these data show that our results cover the majority of the possible spectrum for responsiveness. It is obvious that the matrix derived from these data allows classification of genes by virtue of response patterns. Furthermore, the results demonstrate that patterns of response are diverse and that condition-specific response patterns exist; therefore, our findings implicate multiple mechanisms in diet- and diabetes-responsive gene regulation in the placenta.

**Table 1:** : Patterns of statistical significance of placental gene responses in comparisons between conditions.

Comparison	Chow vs. Breeder	Chow vs. Breeder	Normal vs. Diabetic	Normal vs. Diabetic	
Condition	Normal	Diabetic	Chow	Breeder diet	
Pattern #					Gene Names
1	−	−	−	−	
2	**+**	−	−	−	Usp24, Adamts6, Thbs2, Frem1
3	−	**+**	−	−	Csf2rb
4	−	−	**+**	−	
5	−	−	−	**+**	
6	**+**	**+**	−	−	Tm9sf1, Ermp1, Ptgs2
7	−	**+**	**+**	−	Atoh8, Mpzl2, Pcsk5, Spink8, Tpbpb
8	−	−	**+**	**+**	Lpl
9	**+**	−	−	**+**	Crct1, Mmp1a
10	**+**	−	**+**	−	Ptges2
11	−	**+**	−	**+**	9130005N14R, Il17b, Thsd4
12	**+**	**+**	**+**	−	
13	−	**+**	**+**	**+**	Tgfßi
14	**+**	−	**+**	**+**	Ankrd2, Pappa2, Pla2g5, Prl5a1, Slc6a4
15	**+**	**+**	−	**+**	Kcnk2, Mmp13, Hpgd, Cyp1a1,
16	**+**	**+**	**+**	**+**	Mmp15, Rassf4, Pfpl, Spi16

Comparisons were made for each gene between placentas from chow- and breeder diet-fed normal dams, between placentas from chow and breeder diet-fed diabetic dams, between placentas from normal and diabetic dams fed chow, and between placentas from normal and diabetic dams on breeder diet. Statistical significance by two-tailed t-test for any one comparison is indicated as "+" for comparisons where P is <0.05 (significant), and "−" for P>0.05 (not significant). The response pattern for each gene was determined by virtue of the distribution of significances in these 4 comparisons. vs.  =  versus.

**Figure 5:**
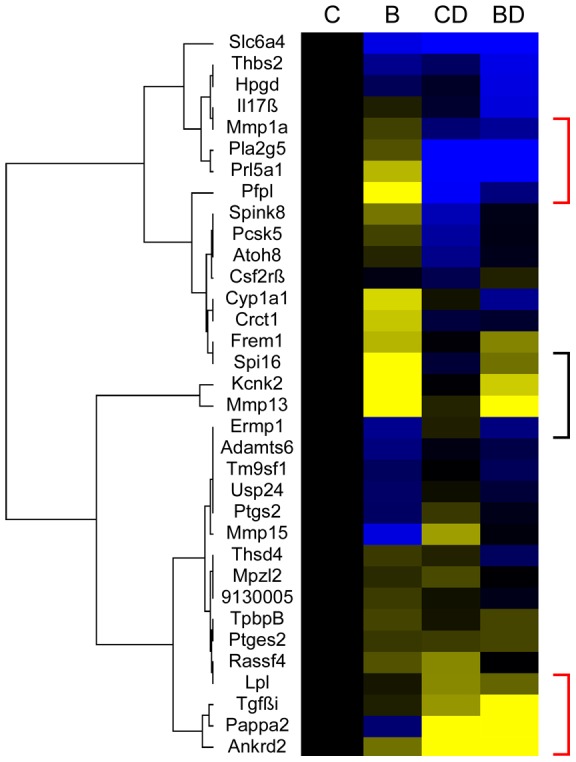
Cluster analysis of diet- and diabetes-responsive gene expression in the placenta. The “fold-change” results for each group and gene were clustered in Cluster 3 and plotted in TreeView as a heat map, with blue representing increased and yellow decreased expression, with black representing no change. The genes most indicative of exposure to diet or diabetes are highlighted by brackets (red for diabetes, black for diet). C: chow diet; B: breeder diet; CD: chow diet, diabetic; BD: breeder diet, diabetic.

This is also evident when the magnitude of changes is considered. Figure 5 displays a hierarchical cluster analysis, where the intensity of color indicates the magnitude of change (blue for increased, yellow for decreased expression relative to the chow diet condition). Genes where diabetes explains more than 50% of the experimental variation -as per Table 1- cluster together as increased (blue) or decreased (yellow) respectively, as indicated by the red brackets. Two of 4 genes where diet explains more than 50% of the variation also cluster together (black bracket). Additional clusters with smaller magnitude of differences in gene expression levels by metabolic and diet condition also appear in this analysis. This provides further evidence in support of the argument for multiple mechanisms. The distinct clustering of genes that respond to either diet or diabetes implies that these genes by themselves can indicate exposure, based upon their expression levels and change from the control.

### Interactions of diet and diabetes on gene expression in the placenta

We then attempted to determine the “strength” of effects of diet and diabetes on gene expression, and potential interactions of both conditions, by performing 2-factor ANOVA statistical tests. These revealed statistically significant interactions between both modalities for only a fraction (11 of 34 = 32.3%) of the genes (Table 2). The extent of variation in placental gene expression explained by the interaction of both factors, metabolic status x diet, varies, with decreasing importance for expression of *Rassf4*, *Cyp1a1*, *Mpzl2*, *Thsd4*, *9130005N14R*, *TpbpB*, *IL17b*, *Ankrd2*, *Pfpl*, *Prl5a1*, and *Mmp13*. Of these, *Cyp1a1*, *TpbpB*, *IL17b*, *Ankrd2*, *Pfpl*, and *Prl5a1* are more responsive to diabetes (with increasing importance of the diabetes factor), and *Mmp13* is more responsive to diet. The greater number of diabetes-responsive genes in this group is likely a reflection of our strategy to select genes from a microarray comparison between diabetic and control placentas [10]. It is noteworthy that the direction of interactions is diverse within this group, as is the relative contribution of metabolic status or diet, providing further support for the proposition that multiple mechanisms of regulation could be involved.

**Table 2:** : Fraction (n %) of variation in gene expression between modalities explained by each factor, and their interaction.

	Interaction	p-value	Diet	p-value	Diabetes	p-value
Gene name						
9130005N14R	**24.77**	**0.0117**	0.54	0.6800	10.24	0.0800
Adamts6	4.41	0.2591	**29.66**	**0.0069**	0.59	0.6751
Ankrd2	**9.73**	**0.0196**	0.22	0.7049	**59.81**	**<0.0001**
Atoh8	2.17	0.4048	12.28	0.0563	**25.70**	**0.0083**
Crct1	8.99	0.0730	**11.99**	**0.0409**	**28.83**	**0.0029**
Csf2rb	13.59	0.0790	6.50	0.2160	0.32	0.7790
Cyp1a1	**37.34**	**0.0001**	0.26	0.6977	**28.76**	**0.0005**
Ermp1	0.45	0.5561	**71.20**	**<0.0001**	1.88	0.2470
Frem1	0.36	0.7392	**36.42**	**0.0027**	0.79	0.6203
Hpgd	2.77	0.2400	**42.65**	**0.0001**	**16.37**	**0.0083**
Il17b	**13.82**	**0.0226**	5.00	0.1527	**35.90**	**0.0007**
Kcnk2	5.77	0.0618	**57.99**	**<0.0001**	**6.74**	**0.0451**
Lpl	3.98	0.1660	0.04	0.8904	**57.49**	**<0.0001**
Mmp13	**4.35**	**0.0250**	**79.55**	**<0.0001**	1.27	0.2054
Mmp1a	4.65	0.1549	0.82	0.5420	**51.99**	**<0.0001**
Mpzl2	**27.70**	**0.0104**	2.06	0.4491	1.06	0.5866
MT2-Mmp	0.21	0.7556	**31.56**	**0.0009**	**26.25**	**0.0021**
Pappa2	1.30	0.1800	**5.17**	**0.0120**	**83.52**	**<0.0001**
Pcsk5	1.07	0.5650	**14.36**	**0.0445**	**22.10**	**0.0150**
Pfpl	**6.36**	**0.0221**	**20.21**	**0.0003**	**52.76**	**<0.0001**
Pla2g5	0.92	0.4501	1.49	0.3380	**66.72**	**<0.0001**
Prl5a1	**5.73**	**0.0441**	4.20	0.0805	**65.26**	**<0.0001**
Ptges2	7.57	0.1308	**13.82**	**0.0459**	**17.61**	**0.0261**
Ptgs2	0.10	0.8405	**34.99**	**0.0011**	**17.11**	**0.0145**
Rassf4	**49.37**	**0.0002**	2.37	0.3243	1.75	0.3958
Slc6a4	1.39	0.3485	**22.50**	**0.0010**	**46.04**	**<0.0001**
Spi16	0.75	0.4013	**67.44**	**<0.0001**	**11.55**	**0.0030**
Spink8	0.15	0.8126	**18.92**	**0.0151**	**27.31**	**0.0046**
Tgfbi	2.92	0.2169	**9.28**	**0.0341**	**51.90**	**<0.0001**
Thbs2	0.51	0.6974	**22.47**	**0.0168**	10.93	0.0840
Thsd4	**26.94**	**0.0070**	3.48	0.2932	9.89	0.0838
Tm9sf1	0.07	0.8660	**49.76**	**0.0002**	0.00	0.9949
Tpbpb	**15.60**	**0.0428**	1.89	0.4606	**15.89**	**0.0411**
Usp24	0.64	0.6553	**33.89**	**0.0035**	3.36	0.3109

Statistical significance was evaluated by two-way repeated measures ANOVA, followed by post-hoc Bonferroni correction for multiple testing. Significance where P<0.05 is indicated by bold font.

Although statistical interaction of exposure conditions was not detected for 23 genes, 12 of these genes were nonetheless regulated by *both* diabetes and diet; given the lack of statistically detectable interactions, we presume that the effects of both factors are independent of each other. We can further classify the response patterns of these genes, as predominantly regulated by diet (in descending order of explanatory value of the diet factor): *Spi16*, *Kcnk2*, *Hpgd*, *Ptgs2*, *Mmp15*, or as predominantly regulated by diabetes: *Pappa2*, *Tgfßi*, *Slc6a4*, *Crct1*, *Spink8*, *Pcsk5*, and *Ptges2* (again in descending order). Finally, genes for which only one factor is explanatory are either responsive to diabetes only: *Pla2g5*, *Lpl*, *Mmp1a*, and *Atoh8* fall into this group, or responsive to diet only: this subset is constituted by *Ermp1*, *Tm9sf1*, *Frem1*, *Usp24*, *Adamts6*, and *Thbs2*. Neither an interaction nor any of the single factors had significant explanatory value for expression levels of *Csf2rb*.

**Figure 6:**
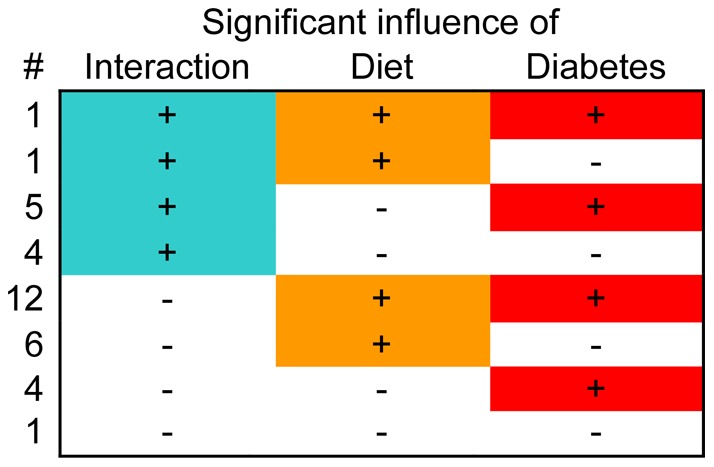
Interactive and independent action of maternal diet and diabetes on gene expression in the placenta. The influence (color) or lack thereof (empty field) of each factor, and their interaction was taken from Table 2, with highlighting for interactions in green, for diet in orange, and for diabetes in red. The number of genes in each category is given on the left.

The patterns of interactions in response to the environmental factors diet and diabetes are schematically depicted in Figure 6. Our finding that diabetes and diet act independently on expression of the majority (67.6%) of these genes indicates that they would be excellent biomarkers to detect exposure to different diets, with or without hyperglycemia. For example, high expression of Pla2g5 and/or Prl5a1, as well as low expression of Ankrd2 and/or Pappa2, indicates exposure to diabetes, while low expression of Kcnk2 and/or Mmp13 indicates exposure to breeder diet. Together with other genes that respond more strongly to diet than diabetes, our results therefore identify biomarkers for adverse diet exposure in early stages of placenta formation. These molecular markers will therefore be most useful in helping to define the potentially detrimental components in the breeder diet, or any other diet.

## Discussion

We here report that maternal diet affects placental growth and gene expression in diabetic pregnancies. In the context of maternal diabetes, the diet recommended specifically for breeding and lactating mice was associated with reduced fetal size [9] and decreased placental size (as measured by weight), indicating that there is a specific interaction of diet with the diabetic condition on placenta growth. Conceivably, this could potentially be linked to reduced consumption of the diet. When fed breeder diet, diabetic dams had reduced weight gain compared to normal dams on this diet, but diabetic chow-fed dams also had reduced weight gain. Thus, reduced weight gain is attributable to the metabolic condition, independent of diet. Interestingly, normal dams gained similar amounts of weight with either diet, indicating that differences in caloric content of the diets had no noticeable influence on maternal weight under normal conditions. With the metabolic derangements of maternal diabetes, however, the difference between the two diets had a strong influence on the differential decrease in placental weight: in comparison to chow-fed diabetic dams, the breeder diet-fed diabetic dams had significantly reduced placenta growth.

Litter size is known to be inversely correlated to placenta size [12], [13]. However, in our experiments, litter size did not significantly differ between diabetic pregnancies when the dam was fed breeder diet compared to the other conditions [9]. Thus, litter size is unlikely to serve as an explanation for reduced placenta growth. It should be kept in mind, that the present study included only placentas that were associated with morphologically normal embryos; analyses on placentas associated with abnormal embryonic development in diabetic pregnancies have yet to conducted.

**Table 3:** : Primers for used for quantitative real-time PCR assays.

Gene symbol	Accession #	Forward primer – sequence	Position	Reverse primer – sequnce	Position	Exon-exon boundary	AE
9130005 N14Rik	NM_026667	GAAGTCACTGCACGCTGCAT	1438–1457	CTGTGGTTAATTTTATCAGAACTCTTGCT	1555–1527	yes	1.89
Adamts6	NM_001081020	GGTCAGGTGTATGATGCTGATGA	2138–2160	AGCTCTCTACACACTTCCCCATATTT	2226–2201	yes	1.90
Ankrd2	NM_020033	GACACCAACGTGAGAGACAAGCT	644–666	CACAATCTCCACGTGTCCAGTAC	718–696	yes	1.93
Atoh8	NM_153778	GGGCGAGCCAAGAAACG	1356–1372	CTGGTGGTCCCAGCTTTCTC	1459–1440	yes	1.96
Crct1	NM_028798	CTTCTGCCTAGCAGGTGTCAAGT	4–26	CGGCGTTTGTCAAGATGAATTAG	91–69	yes	1.88
Csf2rb	NM_007780	CTTCGCTTTGGCTGTGTCTCT	1615–1635	GACCTTTACCTCCATCCTGGAA	1717–1696	yes	1.91
Cyp1a1	NM_009992	AAGAGATACAAGTCTGAATGGCTTCTATATC	1222–1252	AGGCCGGAACTCGTTTGG	1336–1319	yes	1.86
Ermp1	NM_001081213	AGTGCCGTCTGGGTAGTTTTTC	1704–1725	GCCCGTAAAGATATGGGATAAACA	1833–1810	yes	1.95
Frem1	NM_177863	ACAAAAGCGGCGGTGAAA	5834–5851	GAAGTGGTGAGCGAGGATGAG	5973–5953	yes	1.91
Hpgd	NM_008278	GACCTATCTTGGTTTGGATTACATGAG	369–395	GAGCCCTGCTAATGAAGACATATTG	453–429	yes	1.90
Il17b	NM_019508	GCCAAGAAGAAATGTGAAGTCAATCT	287–312	GGGTCGTGGTTGATGCTGTA	375–356	yes	1.97
Kcnk2	NM_010607	GTGGTTATCACTCTGACGACCATT	778–801	AGGCTTGTAGAAGTCCAGATATTCAAT	861–835	yes	1.89
Lpl	NM_008509	TTATCCCAATGGAGGCACTTTC	958–979	CCACGTCTCCGAGTCCTCTCT	1043–1223	yes	1.88
Mmp1a	NM_032006	AGGCAGGTTCTACATTCGGGTAA	941–963	TGGCCAGAGAATACCTATTAAATTGA	1013–988	yes	1.95
Mmp13	NM_008607	AATCTATGATGGCACTGCTGACAT	478–501	GTTTGGTCCAGGAGGAAAAGC	595–575	yes	1.84
Mmp15	NM_008609	ATGCAGCCTACACCTACTTCTACAAG	2085–2110	CCATGAAGTCCCGCAGGAT	2192–2174	yes	1.89
Mpzl2	NM_007962	GGGCGGACAGTGCTGATAAA	728–747	TCCACAAAAACAGAGACCTTGTTTC	815–791	yes	1.92
Pappa	NM_021362	GAGTGCAAGTTGGGCTTCTTAAA	10045–10067	AGAGACCCAAGAAAGCAACTCAA	10130–10107	no	1.93
Pcsk5	NM_001163144	GCAAGGGCGGGTTAAGTCTT	2303–2322	TGGCAGTCGTGACCATTGA	2384–2366	yes	1.89
Pfpl	NM_019540	AACCAGTGTTGTGGAGACTCCAA	2583–2605	AATTCTAACTGTGCAGCAGACAGAAA	2699–2674	no	1.89
Pla2g5	NM_001122954	CCCAAGGATGGCACTGATTG	460–479	TCCGAATGGCACAGTCTTTTT	541–521	yes	1.88
Prl5a1	NM_023746	CAAACAACAAAAGGAAGGCTGAA	346–368	GCAGCCAGCATTCTAATTGTCA	418–397	yes	1.89
Ptges2	NM_133783	TGCCATGTACCTCATCAGCAA	1228–1248	AGAGGTCTACCCGTACATCATCCT	1298–1275	yes	1.90
Ptgs2	NM_011198	CAACAACTCCATCCTCCTGGAA	1285–1306	GAGGCCTTTGCCACTGCTT	1410–1392	yes	1.86
Rassf4	NM_178045	GACCAACGTCCGGGTTAACA	667–686	CCAGACTCATGGACGGTGTAGAG	786–764	yes	1.89
Slc6a4	NM_010484	TGGCCATCAGCCCTCTGT	1813–1830	TGTATTGGAAAAGCCGGAGTTG	1893–1872	yes	1.83
Spi16	U96702	TGACCGCCCATTCCTTTTC	363–381	GAAGAGAACCTGCCACAGAACAA	434–412	no	1.95
Spink8	NM_183136	GGCCAGCTCAGTGTGGACTT	171–190	AAGCTCCCCGGTCATGTG	243–226	yes	1.98
Tgfbi	NM_009369	CGGTGTGGTCTATGCCATCA	1930–1949	GCTGACGCCTGTTTGAAGATT	2040–2020	yes	1.69
Thbs2	NM_011581	CAGGTGGCACCTGATTCACA	3693–3712	GCGTAGGTTTGGTCATAAATTGG	3800–3778	yes	1.81
Thsd4	NM_172444	CAGCTTCCTGGCACATTGC	450–468	GAGCCCCTGAATACGTCAAAAG	587–566	no	1.97
Tm9sf1	NM_028780	GAGCCACTTCTACCGGCAAA	1461–1480	AAGTCAGGAAGAAAGGCACAGAGA	1552–1529	yes	1.95
Tpbpb	NM_026429	CAGAGAGTGGCGATGGGTTT	406–425	TGTTTCACTCGTTGCCTAACTTCA	487–464	yes	1.88
Usp24	NM_183225	CATCAGTTCAGTCTCCATATAGATCAACA	2972–3000	AATAGTTCGTGGGACAGAGTAGAAGTC	3087–3061	yes	1.80

Primer sequences and positions on the reference sequence are given. Where possible, primers were designed to span an exon-exon junction so as to avoid amplification from potentially contaminating DNA. Amplification efficiencies were calculated from the actual PCR runs as described before.

Interestingly, maternal glucose levels were higher at the start of pregnancy when diabetic dams consumed breeder diet, averaging 349.65±97.79 mg/dL compared to 306.23±81.81 mg/dL in diabetic dams consuming the chow diet. By the time of sacrifice, maternal glucose levels exceeded the upper limit of the meter (600 mg/dL) in 9 out of 53 dams on chow diet, and in 29 out of 45 dams on breeder diet (we therefore cannot estimate average levels for the whole group); measurable blood glucose levels in the remaining dams were 457.50±90.61 mg/dL in chow-fed (n = 26), and 506±82.13 mg/dL in breeder diet-fed diabetic dams (n = 16), respectively (difference is not statistically significant). Also, if we consider the difference between pre-pregnancy glucose levels and those at sacrifice [9] as an indicator of diabetes severity, there was only a weak relationship to placenta growth in diabetic pregnancies, regardless of diet. Dam weight at copulation was 22.46±1.8 g (n = 27) for breeder diet-fed diabetic dams, which was approximately 1g less than normal dams (23.5±2.1 g; n = 48) on breeder diet. However, compared to diabetic dams on chow diet, the weights, and the weight gains, of breeder diet-fed diabetic dams were indistinguishable throughout pregnancy. Thus, we consider it unlikely that solely the degree of maternal hyperglycemia, or maternal size or weight gain, could have been responsible for the reduced placenta size in breeder diet-fed diabetic pregnancies. Because our data do not implicate maternal factors other than diet in the reduced growth of placentas in diabetic pregnancies, we therefore conclude that diet is the major factor influencing placenta growth in this model.

The gene expression profiles indicate that breeder diet does not simply exacerbate the detrimental effects of maternal diabetes, but that it has distinct effects. While gene expression is clearly misregulated in diabetic placentas, the different diets influence the magnitude and direction of changes, and exert their effects on specific sub-sets of genes. Except for Tgfßi and Il17ß, where gene expression levels in diabetic placentas could be interpreted to correlate with blood glucose levels (magnitude of change is greater in the breeder diet-fed group than in the chow-fed), all other patterns are indicative of interaction of diet and diabetic state, in additive manner, and often also in opposite directions (see Figure 4). Examples to illustrate additive effects are Thbs2, Ptgs2, Hpgd, Slc6a4, Mmp15, Pfpl, and Spi 16; examples for opposite direction of the diet effect in normal compared to diabetic dams are Crct1, Mmp1a, Atoh8, Cyp1a1, Pappa2, Ankrd2, Prl5a1, and Rassf4. In addition, our results reveal several genes that can serve as indicators of diet exposure in the absence of and regardless of maternal diabetes, such as Usp24, Adamts6, Frem1, Tm9sf1, Emp1, Ptgs2, Kcnk2, Mmp13 and Mmp15. These patterns, and particularly those of opposite interactions, imply that the adverse effect of breeder diet on placenta growth acts through mechanisms other than hyperglycemia alone. Thus, we have identified diet-dependent targets, of which some interact with maternal diabetes in regulating gene expression in the placenta at midgestation.

Less clear at the moment is how these molecular alterations translate into reduced placental growth. We have previously shown that spongiotrophoblast growth is reduced under conditions of diabetic pregnancy, and the labyrinth also remains smaller [10]. We also reported reduced levels of *Ascl2* (achaete-scute complex homolog 2), which is normally expressed in and required for growth of spongiotrophoblasts and the labyrinth [14]. However, *Ascl2* is only regulated by diabetes, not by diet (unpublished observations), and thus cannot account for the greater extent of growth reduction of diabetic placentas in breeder diet-fed dams. More plausible candidates are therefore those genes that exhibit a response to both diabetes and diet. Among those diet-dependent genes that could contribute to altered placental growth are genes known to play a role in inflammation. In this context, the upregulation by diet of genes encoding thrombospondin-domain containing proteins (*Adamts6*, *Thbs2*, *Thsd4*) and the downregulation by diet of proteinase inhibitors *Spink8* and *Spi16* are noteworthy. Similarly, diet modulates the expression of genes encoding enzymes that are involved in eicosanoid metabolism, such as *Prostaglandin endoperoxide synthase 2* (*Ptgs2/Cox-2*), *Hydroxyprostaglandin dehydrogenase* (*Hpgd*) and *Cyp1a1*. The enzymes encoded by these genes are involved in production and metabolism of prostaglandin E2, and our working model is that under conditions hyperglycemia and adverse diet, enzymes catalyzing PGE_2_ degradation are elevated to levels where they create a functional PGE_2_ deficiency, through increased catabolism of PGE_2_. Prostaglandin E2 has been shown to stimulate trophoblast migration [15], [16], [17] and cellular invasive behavior [18], [19]. Conversely, in experimental animals with reduced PGE_2_ levels, cell migration is reduced [20], [21], [22]. Our previous histological analysis of diabetic placenta revealed aberrant trophoblast migration and reduced growth of the spongiotrophoblast layer [10], which is consistent with impaired PGE_2_ signaling. Another role of PGE_2_ is inactivation of Natural Killer Cell activity in the decidua [23]. Intriguingly, we observe high NK cell accumulation in the diabetic placenta [10], again consistent with PGE_2_ deficiency. A second indication for the involvement of inflammatory pathways in reduced placental growth is the upregulation by breeder diet, at least in the diabetic state, of *IL17b*, a member of the pro-inflammatory IL17 cytokine family. The role of inflammatory pathways in aberrant placenta development as a consequence of diabetes or diet warrants further investigation. Also intriguing is the upregulation by breeder diet of *Ubiquitin-specific gene 24* (*Usp24*) and of *Endoplasmic reticulum metallopeptidase 1* (*Ermp1*), which could be reflective of altered protein processing. Taken together, we detect diet influences on genes with plausible roles in stress responses and inflammation; further studies will be required to demonstrate a functional relation to placental development and growth.

It is noteworthy that our -admittedly short- list of 33 diet-responsive genes does not overlap with the gene repertoire changes reported for placentas from protein restricted FVB dams at E17.5 [24], or for a comparison of low fat and high fat content diets in NIH Swiss dams at E12.5 [25]. Non-congruency could be explained by the different times of sampling, use of different diets, and the fact that our breeder diet (Purina 5015) was used as the control diet in the second paradigm, which also encompasses a strain difference. Yet, the most important feature in our study is the presence of maternal diabetes as a second environmental factor that produces the sensitizing condition under which the adverse effects of breeder diet on placental growth, and the novel interactions of diet and diabetes on gene expression that we have identified here, are revealed.

The molecular mechanisms through which diet affects the regulation of genes with altered expression levels are unknown. To date, regulatory elements that confer placenta-specific expression have not been identified for any of the diet targets our work uncovered. Similarly, it is unknown whether microRNAs or other epigenetic mechanisms may be involved. Changes in cellular composition, namely increased frequency of cells expressing the respective gene, appear to be responsible for the increased expression of the *Serotonin transporter* (*Slc6a4*) and *Cyp1a1* genes in diabetic placenta [10]. Yet, we do not currently know to what extent, and if so, how any particular diet affects the cellular make-up of the placenta.

Both the chow, as well as the breeder diet, are formulated to be replete for minerals and micronutrients, but they differ in macronutrient composition. In particular, protein content is higher in the chow diet, while fat is enriched in the breeder diet. From our results, it appears that placental cells can detect this difference, likely through nutrient sensing mechanisms [26], [27]. The mTOR system plays a prominent role in nutrient sensing, and it has recently been shown to be present in placenta [28], [29]. Although we did not obtain evidence from our microarrays for altered expression of genes in the mTOR pathway in diabetic placenta [10], it would be expected to play a role in response to different diets. It should also be kept in mind that, although “defined” in their composition, both diets are manufactured from natural ingredients, such as soybean-derived products and fish meal, the quality and molecular composition of which can be variable. In this regard, it is important to note that in normal pregnancies, *Cyp1a1* expression is decreased by breeder diet, providing evidence that this diet is not simply contaminated with unidentified toxins. Nonetheless, assays with purified ingredient diets are necessary to determine which of the components in the breeder diet is/are responsible for deficient placental development. The 33 diet-responsive genes we have identified in the present study will be most valuable in monitoring exposure to different nutritional conditions and perturbations.

Taken together, our results demonstrate that maternal diet modulates placental gene expression and growth, with a concomitant effect on fetal growth [9]. Because deviations from normal birth weight are linked to adult disease risk, the placental alterations we find in response to diet and diabetes may have important implications for developmental programming of susceptibility to disease later in life [30], [31].

## Methods

### Animals, induction of diabetes, and diets

Mice of the FVB inbred strain were obtained from The Jackson Laboratories (Bar Harbor, ME) at the age of 5–6 weeks old and were accomodated to the animal facility for one week before any experimentation. Diabetes was induced in female mice by two injections of Streptozotocin within a week as previously described [9], [10], [11]; when their glucose levels exceeded 250 mg/dL, mice were set up for breeding, but no earlier than at least 7 days after the last STZ injection. The day of detection of a vaginal plug in the morning was termed gestational day 0.5 (E0.5). Details for glucose and weight measurements of the dams used in this study have been published [9]. All animal experimentation was done with approval by the Pennington Biomedical Research Center Institutional Animal Care and Use Committee, in accordance with ‘Principles of laboratory animal care’ (NIH publication no. 85–23, revised 1985; http://grants1.nih.gov/grants/olaw/references/phspol.htm) and applicable federal and state regulations. Diets were obtained from LabDiet/Purina Mills International (Richmond, IN). The standard diet was Purina 5001 (chow) until the females were placed into two groups for the experiments at the age of 8 weeks: one group was fed Purina 5001 (chow) and the other group received Purina 5015 (breeder diet) from thereon. The manufacturer states that Purina 5001 has a physiological fuel value of 3.36 kcal/g, with 28.5% of calories are derived from protein, 13.5% from fat, and 58% from carbohydrates, and Purina 5015 a physiological fuel value of 3.83 kcal/g, 19.8% of calories are derived from protein, 25.3% from fat, and 54.8% from carbohydrates (http://www.labdiet.com/rodent_diet.html).

### Placenta isolation

At designated days, uterine horns were dissected out, and pairs of placentas and embryos were isolated. Placentas included embryo-derived and maternal tissue, and were dissected in PBS, briefly blotted on tissue paper to remove excess liquid, and then they were weighed [10]. Only placentas associated with morphologically normal embryos were used for this study.

### Quantitative real-time PCR

Details of the quantitative real-time PCR (Q-RT-PCR) method have been described elsewhere [10], [32]. Primer pairs used in this study are listed in Table 3. The data analysis for the PCR results was also previously described [11], including normalization to *Polymerase epsilon 4* (*Pol*ε*4*) expression, and calculation of amplification efficiencies and fold changes. Statistical tests were done on ΔC_t_ values for a group size of 6 per modality. All assays were performed on at least 6 placentas, each from an independent pregnancy, for each metabolic condition and diet modality.

### Statistical evaluation

Results were evaluated for statistical significance by using two-tailed T-tests for pairwise comparisons. P-values smaller than 0.05 were considered statistically significant. For the interaction analyses, two-factor repeated measures ANOVA was applied, with Bonferroni post-hoc correction for multiple testing, as implemented in GraphPad Prism version 4.

Cluster analyses were performed in Cluster 3 (http://rana.lbl.gov/EisenSoftware.htm) by hierarchical clustering of genes using the fold-change data depicted in Figure 4, without any data filtering or adjustments, based on Euclidian distance, with complete linkage. Visualization was done with Java TreeView (http://jtreeview.sourceforge.net/).
